# Protected fish spawning aggregations as self-replenishing reservoirs for regional recovery

**DOI:** 10.1098/rspb.2023.0551

**Published:** 2023-05-10

**Authors:** Brian C. Stock, Andrew D. Mullen, Jules S. Jaffe, Allison Candelmo, Scott A. Heppell, Christy V. Pattengill-Semmens, Croy M. McCoy, Bradley C. Johnson, Brice X. Semmens

**Affiliations:** ^1^ Scripps Institution of Oceanography, University of California, San Diego, La Jolla, CA 92093, USA; ^2^ Institute of Marine Research, Nye Flødevigveien 20, 4817 His, Norway; ^3^ Reef Environmental Education Foundation, Key Largo, FL 33037, USA; ^4^ Central Caribbean Marine Institute, N Coast Road E Box 37, Little Cayman KY3–2501, Cayman Islands; ^5^ Department of Fisheries, Wildlife, and Conservation Sciences, Oregon State University, Corvallis, OR 97331, USA; ^6^ Department of Environment, Cayman Islands Government, Grand Cayman KY1–1002, Cayman Islands; ^7^ School of Ocean Sciences, Bangor University, Menai Bridge LL59 5AB, UK

**Keywords:** larval dispersal, plankton imaging, fish spawning aggregation, recruitment, Nassau grouper

## Abstract

Dispersal of eggs and larvae from spawning sites is critical to the population dynamics and conservation of marine fishes. For overfished species like critically endangered Nassau grouper (*Epinephelus striatus*), recovery depends on the fate of eggs spawned at the few remaining aggregation sites. Biophysical models can predict larval dispersal, yet these rely on assumed values of key parameters, such as diffusion and mortality rates, which have historically been difficult or impossible to estimate. We used *in situ* imaging to record three-dimensional positions of individual eggs and larvae in proximity to oceanographic drifters released into egg plumes from the largest known Nassau grouper spawning aggregation. We then estimated a diffusion–mortality model and applied it to previous years' drifter tracks to evaluate the possibility of retention versus export to nearby sites within 5 days of spawning. Results indicate that larvae were retained locally in 2011 and 2017, with 2011 recruitment being a substantial driver of population recovery on Little Cayman. Export to a nearby island with a depleted population occurred in 2016. After two decades of protection, the population appears to be self-replenishing but also capable of seeding recruitment in the region, supporting calls to incorporate spawning aggregation protections into fisheries management.

## 1. Background

Recruitment, i.e. survival of offspring to the subadult stage, is the principal driver of natural variability in adult abundance for many marine organisms [1–3]. For species with limited adult movement, as is common for tropical reef fish, the proportion of larvae that are exported from a spawning site versus retained locally is pivotal because it determines the appropriate spatial scale of management [4–6]. If retention dominates, population dynamics are relatively insensitive to the periodic arrival of external recruits. This simplifies management, as local protections directly benefit local populations [7]. If export dominates, local actions have less effect and population-scale impacts of fishing depend on the connectivity between locations. Thus, larval dispersal matters for both population forecasting and the spatial design of marine reserves, for example, in determining the extent that larval export from a reserve benefits neighbouring fished areas [5,8–12]. This is particularly important for the recovery of overfished, aggregating species like Nassau grouper (*Epinephelus striatus*), formerly one of the most important food fish in the Caribbean but now Critically Endangered [12–14]. Many historic Nassau grouper fish spawning aggregations (FSAs) no longer form [12], and region-wide recovery likely depends on the fate of eggs and larvae spawned at, and dispersed from, the few healthy FSAs that remain.

Reef fish larval dispersal was formerly assumed to be long-range [15], but in the last two decades this paradigm has been convincingly overturned by studies showing that recruitment is often local [16–24] and that larvae can detect, orient to and capably swim toward home reefs [16,22,25–28]. Efforts to predict dispersal have evolved from simple advection–diffusion–mortality models [15,29–33] to individual-based models with increasingly high-resolution physics and biological realism [4,34–36]. However, modelling studies are still often based on little to no evidence from direct field observations of egg and larval concentrations because they are difficult to obtain at the necessary spatial and temporal scales. This lack of field data on initial distributions is problematic because small differences in starting location can have large impacts on dispersal, especially near the coast where currents and topography are often complex [37]. Among many methods used to quantify dispersal [4,16], releasing drogued drifters is an attractive option for reef fish that form large spawning aggregations. In these cases, all annual reproductive output may be released over only 2–4 days and from well-defined spatial locations that are consistent across years [38–44].

We released drifters into egg plumes from the large Nassau grouper FSA off the west end of Little Cayman, Cayman Islands over multiple years (2011, 2016 and 2017) as part of the Grouper Moon Project. For two cohorts spawned in 2017, we used an *in situ* plankton imaging system mounted on an undulating towed vehicle to observe the three-dimensional positions of individual eggs and larvae around the drifters up to 36 h after spawning. We used these data to estimate parameters of a three-dimensional diffusion–mortality model and then predicted the concentration of eggs and larvae around previous years’ drifter tracks to evaluate the possibility of retention and export to nearby islands within 5 days of spawning—before larvae develop the swim bladder and fins later used to influence transport [45,46]. Our models predict local retention on spawning nights in 2011, a key year when a large cohort was spawned that subsequently drove population recovery [47,48]. Export to a neighbouring island with a depleted population likely occurred in 2016. In 2017, we directly observed that eggs and larvae spawned at the Little Cayman FSA were transported back to Little Cayman reefs. Thus, the Little Cayman Nassau grouper population appears to be both self-replenishing and capable of seeding recruitment in the region.

## 2. Methods

### (a) Study species: Nassau grouper

Nassau grouper are ecologically, economically and culturally important predatory reef fish in the Caribbean [49]. They form large (historically up to tens of thousands), transient (6–8 days) fish spawning aggregations (FSAs) at highly predictable locations following full moons [44,49–51]. The 2–4 days over which spawning occurs at these FSAs likely represent total annual reproductive output [44]. Overfishing of FSAs has led to dramatic declines throughout the Nassau grouper's range, causing the species to be listed as Critically Endangered by the International Union for Conservation of Nature (IUCN) and Threatened under the United States' Endangered Species Act [13,14]. Seasonal and spatial FSA protections have been broadly instituted, yet populations have failed to recover in many places [43]. Although recovery of the species depends on successful spawning, recruitment, and connectivity between sites, little is known about the necessary conditions for this to occur [12]. The possibility of Nassau grouper larvae settling near the FSA where they were spawned (i.e. self-recruitment) has long been hypothesized but has not yet been documented [40,41,52,53].

Nassau grouper early life history has been described for laboratory-reared collections of eggs at FSAs or induced ovulation of captured females. Eggs are transparent, spherical, 0.86–1.02 mm in diameter, neutrally buoyant at 32‰ salinity, and typically contain a single oil globule [46,54]. Time to hatching is temperature-dependent and takes roughly 24–27 h at 26°C [46,53,55]. Recently hatched larvae are unpigmented and slightly curved around their yolk-sac [46]. At 3–4 days post hatch (dph), larvae begin feeding and gain pigmentation in the eyes and caudal peduncle. By 5–7 dph, larvae exhaust their yolk and oil reserves and will starve if unfed [55]. Larval swimming speed and endurance greatly increase after notochord flexion in related taxa [56,57], which occurs around 5–6.5 mm and 16–20 dph in Nassau grouper [46]. The few measurements of swimming speed for postflexion Serranidae and Epinephelidae larvae indicate that they can swim 25–35 cm s^–1^ at settlement stage [57,58]. Larvae transition from pelagic to juvenile habitat around 35–45 dph and 20–27 mm length [52,59]. Juveniles sexually mature and join the spawning population between 4 and 7 years old [49].

### (b) Study location: Little Cayman FSA

Nassau grouper aggregate to spawn at the southwestern tip of the Little Cayman shelf (electronic supplementary material, figure S1). The shelf slopes very gradually to 30 m depth and then abruptly drops to over 500 m [60]. Spawning has been observed by divers yearly since 2002 [48,50]. The fish form a band along the shelf edge with some individuals scattered nearby on the bottom, with spawning beginning shortly after sunset and lasting about 1 h. Small groups of 10–15 individuals rapidly ascend and release gametes between 20 and 30 m, then return to the bottom. Prevailing winds and currents are westward, with the FSA site on the leeward side of the island. Currents at the site are complex, vary vertically, and range from slack to 3 knots [50].

The Little Cayman Nassau grouper FSA is currently the largest known for the species [14,48,61]. The population declined following heavy FSA fishing in 2001–2002, but was protected in 2003 and has since recovered in numbers, spawning biomass, and size structure [47,48]. Recruitment is highly variable with one particularly strong year class, 4–8× average, spawned in 2011, which drove the recent population increase [47]. In years relevant to this study, the FSA comprised roughly 2100 (2011), 4200 (2016) and 5400 (2017) mature fish [48]. Populations on each of the Cayman Islands are considered distinct, based on acoustic tagging data showing that adult Nassau grouper do not cross the abyssal depth water between islands [60,62,63].

### (c) Drifter deployments

Upon visual confirmation of peak spawning by divers, we released standard drogued surface velocity programme (SVP) drifters to mark egg patches, which track currents at 15 m depth (Pacific Gyre, Inc. and Global Drifter Program). We released 1 or 2 drifters near the end of the evening's spawning on the 2 or 3 nights per year determined to be peak spawning (24–25 Feb. 2011, 25–26 Feb. 2016 and 14–16 Feb. 2017; electronic supplementary material, figure S1). On 15 February 2017, we released five drifters staggered throughout the hour of spawning. Divers signalled to the boat when and where spawning occurred by sending a float to the surface. The drifters then served as visual references for plankton sampling, and we affixed strobes to the drifter floats to enable visual tracking at night.

### (d) *In situ* plankton imaging

We sampled egg patches in 2017 with the TowCam, which consisted of an *in situ* plankton imaging system mounted on an undulating towed platform (Acrobat, Sea Sciences, Inc.; electronic supplementary material, methods, figure S2) [64,65]. The entire system was operated by hand from a 14 m boat by a team of two researchers, allowing relatively low-cost nearshore operations. We continuously sampled eggs spawned on 14 February 2017 for 15 h using the TowCam, returned to the FSA, and followed eggs spawned on 15 February 2017 for 36 h. We towed the TowCam along alternating 1–2 km transects perpendicular and parallel to the drifter trajectories, undulating between 1 and 30 m depth at roughly 3 cycles km^–1^. When the drifters were over the shelf, we limited our vertical sampling to the upper 10–15 m owing to concerns of hitting the reef.

We used a combination of manual and algorithm-based classification to identify *in situ* images as fish eggs (electronic supplementary material, methods). We then classified fish egg images with diameter between 0.87 and 1.20 mm as Nassau grouper eggs, since two methods indicated that this was a reliable metric: measuring Nassau grouper eggs collected by divers 10–30 s after gamete release, and DNA barcoding [66] of fish eggs in plankton samples near drifters (electronic supplementary material, methods, figure S3).

### (e) Plankton sample collection

**Figure 1.**
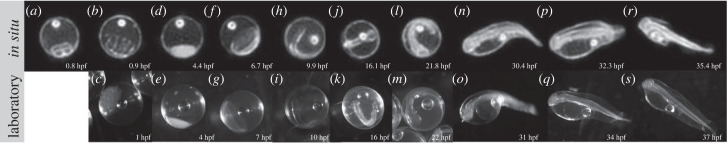
Image sequences of Nassau grouper egg and larval development over 1–37 h post-fertilization (hpf). Top: *in situ* images taken in the egg cloud spawned on 15 February 2017. Bottom: light microscope images of laboratory-reared eggs and larvae collected from the same spawning event. (*a*) Early cleavage period, four-cell stage (less than 1 hpf). (*b,c*) Late cleavage period, regular rows of blastomeres (1 hpf). (*d,e*) Blastula period, yolk cell bulging (4 hpf). (*f,g*) Early gastrula period, blastoderm is an inverted cup rising from animal pole to vegetal pole (7 hpf). (*h,i*) Late gastrula period, rudimentary notochord visible (10 hpf). (*j,k*) Segmentation period (16 hpf). (*l,m*) Near hatching (22 hpf). (*n,o*) Early yolk-sac larvae, notochord curved (31 hpf). (*p,q*) Early yolk-sac larvae, notochord straightening (32.3–34 hpf). (*r,s*) Yolk-sac larvae, notochord straight and yolk reduced in size (35.5–37 hpf). *In situ* image pixels are 22.6 µm × 22.6 µm.

To validate the *in situ* image classification, we collected plankton samples using a variety of methods (electronic supplementary material, methods). On nights of TowCam sampling in 2017, divers collected Nassau grouper eggs by hand-towing a plankton net through the egg cloud during spawning. These eggs were raised to hatch in aquaria at ambient temperature in filtered seawater to generate an image sequence of egg development (figure 1) and determine time to hatching (electronic supplementary material, figure S4). Divers also collected eggs from individual Nassau grouper spawning bursts 10–30 s after gamete release using plastic zipper bags in 2014–2017.

### (f) Three-dimensional diffusion–mortality model

We calculated the observed Nassau grouper egg concentration (no. eggs l^–1^), *Y_i_*, for each minute *i* along the sampling track by dividing egg image counts (min^–1^) by the TowCam volume sampling rate of 264 l min^–1^. We then estimated horizontal diffusivity and mortality for the two 2017 cohorts by fitting these data to a simple three-dimensional model of advection, diffusion and mortality, described below.

We assumed that the drifters captured the horizontal advection and there was no vertical current shear, subtracted the locations of the drifter centroid from the egg concentration locations, and removed advective terms. We also assumed continuity (i.e. seawater was incompressible), constant mortality in space and time, and separability of vertical and horizontal diffusion. These assumptions allowed us to model the egg concentration at depth *z* and time *t*, *C*(*z*, *t*), independently from mortality, *μ*, and the concentration in horizontal space, *C*(*x*,*y*,*t*), and then multiply the results to get the concentration at any point in three-dimensional space and time:

C(x,y,z,t)=C(z,t)×C(x,y,t).

### (g) Vertical diffusion

To model vertical diffusion, we used a particle-tracking (random-walk) model [67]. We set the initial particle distribution as N(μ=26.4,σ=3) to match the observed depth distribution of the 14 February 2017 cohort in the first hour (figure 3*a*). We simulated 10 000 particles, updating depths at time steps, Δt=10 s, according to:

$$z_{n+1}=z_{n}+\frac{\partial K_{z}(z_{n})}{\partial z}\Delta t+R{\left(\frac{2K_{z}(z_{n}+1/2(\partial K_{z}/\partial z)\Delta t)}{r}\right)}^{1/2}+w_{s}\Delta t,$$

where *R* is a random process with mean zero and variance *r*, *w_s_* is the egg floating speed and *K_z_*(*z*) is the vertical diffusivity. *K_z_*(*z*) decays with depth as:

$$K_{z}(z)=\;K_{0}e^{-z/z_{\mathrm{MLD}}},$$

where *K*_0_ is diffusivity at the surface and *z*_MLD_ is the mixed layer depth. We took the two-dimensional kernel density estimate of the particle distributions to be *C*(*z*,*t*), the egg concentration at depth *z* and time *t*. For model parameterization and fitting details, see electronic supplementary material, methods.

### (h) Horizontal diffusion and mortality

We allowed for anisotropic horizontal diffusivity and assumed that diffusivity was constant in each direction following Fick's Law [68], where the diffusive flux, *J_x_*, is proportional to the concentration gradient, i.e. $J_{x}=K_{x}(\partial C/\partial x)$. Alternative models of diffusion may be more appropriate in general, but assuming Fickian diffusion was justifiable here because the size of the egg patch varied much less than one order of magnitude over the course of our observations (500–1500 m) [68]. Assuming an instantaneous point release at 19.00 local time, the horizontal egg concentration, *C*(*x*,*y*,*t*), is given by [32] and [30]:

$$C(x,y,t)=\;\frac{1}{4\pi t\sqrt{K_{x}K_{y}}}e^{\left(-(x^{2}/4K_{x}t)\;-\;(y^{2}/4K_{y}t)\;-\;\mu t\right)},$$

where *K_x_* and *K_y_* are diffusivity in the *x* (east–west) and *y* (north–south) directions and *μ* is the mortality rate. However, instead of assuming eggs were evenly distributed throughout the water column as in [32], we multiplied the horizontal concentration, *C*(*x*,*y*,*t*), by the vertical concentration, *C*(*z*,*t*), to get the egg concentration at any given *x*, *y*, *z* and *t*.

### (i) Statistical model fitting

We then fitted the observed egg concentration in minute *i*, *Y*(*x_i_*, *y_i_*, *z_i_*, *t_i_*), with a negative binomial generalized linear model (GLM) using *C*(*x_i_*,*y_i_*,*z_i_*,*t_i_*) as the expected mean:

$$Y(x_{i},y_{i},z_{i},t_{i})\;∼\;\mathrm{NB}(\mu =e^{\beta C(x_{i},y_{i},z_{i},t_{i})}).$$

We fitted the model using maximum likelihood via the ‘bbmle’ R package [69], and calculated 95% confidence intervals for the parameters *K_x_*, *K_y_* and *μ* using likelihood profiles. In addition to the full model described above, we fitted all nested submodels and evaluated the importance of including each parameter using Akaike's information criterion (AIC). For further details, diagnostics, and parameter estimates, see electronic supplementary material.

### (j) Modelled dispersal in 2011 and 2016

Finally, we used the above model to predict the distribution of eggs and yolk-sac larvae up to 4 dph around drifters released in 2011 and 2016 from the Little Cayman FSA. For the egg stage (0–24 h), we used our estimated egg mortality rate from 14 February 2017 data. After hatching (24 h), we used a reduced mortality rate of *μ* = 0.576 day^–1^, the mean for yolk-sac larvae at similarly high temperatures (mean of two values above 25°C in Fig. 7a and Table 5 in [70]). We approximated the total annual number of eggs spawned by assuming a sex ratio of 1 : 1 and multiplying the number of females [48] and length distribution [47] of the spawning population by fecundity-at-length [71]. We assumed that annual egg production was distributed between two peak spawning nights (40% each) and two minor spawning nights (10% each) based on diver observations [50]. We estimated that the Little Cayman FSA produced 3.9 (3.1–4.9; propagated 95% CI from number of females) billion eggs in 2011 and 9.2 (7.5–11.3) billion eggs in 2016.

## 3. Results

### (a) *In situ* imaging of eggs and larvae

Over two spawning nights in 2017, we collected *n* = 238 184 *in situ* plankton images from the egg plumes. We classified *n* = 2741 images as fish eggs and 82.6% of those (*n* = 2265) as Nassau grouper eggs, in addition to *n* = 47 images as yolk-sac larvae 30–36 h after spawning (electronic supplementary material, methods and figure S3). The *in situ* images provided sufficient resolution to distinguish egg and larval development stages, and these aligned well with images of eggs and larvae collected from the same spawning event and raised in aquaria (figure 1). This confirmed that the drifters tracked eggs and larvae for at least 36 h beyond hatching at 23–26 h (electronic supplementary material, figure S4).

### (b) Observed larval retention

**Figure 2.**
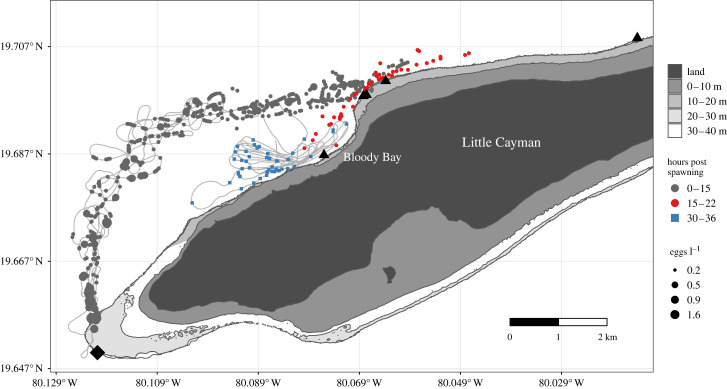
Observed distribution of Nassau grouper eggs and yolk-sac larvae following spawning off the west end of Little Cayman (diamond) on 15 February 2017. Egg image counts min^–1^ were converted to concentration (eggs l^–1^, circle size) based on the imaging volume and frame rate. Circle colour highlights the increased horizontal spread of eggs observed after (hours 15–22, red) versus before (hours 0–15, grey) the drifters grounded. Yolk-sac larvae were observed in Bloody Bay 30–36 h after spawning (blue squares, *n* = 47). Drifter grounding locations are shown as triangles (*n* = 5), and the boat sampling track is shown as a light grey line.

All five drifters released during spawning on 15 February 2017 grounded within 20 h (figure 2). Three of the drifters grounded on a protrusion of the Little Cayman shelf approximately 15 h after spawning, one drifter continued approximately 5 km east along the north edge of the shelf, and the other entered Bloody Bay (figure 2). The upper 30 m of the water column was weakly stratified at the FSA but became well-mixed as the drifters turned east and neared the shelf (electronic supplementary material, figure S5). The horizontal extent of the egg cloud greatly increased when it hit the shelf at approximately 15 h (electronic supplementary material, figure S6), and yolk-sac larvae were observed in Bloody Bay 30–36 h after spawning (figures 1 and 2). A three-dimensional diffusion–mortality model fitted the data well but explained a low percentage of the deviance (11.1%, electronic supplementary material, figure S7 and table S1). Thus, the estimated diffusion and mortality rates from this cohort likely do not apply to spawning events when eggs are transported directly off the shelf, as is typical at the site (electronic supplementary material, figure S1).

### (c) Estimates of diffusion and mortality rates

**Figure 3.**
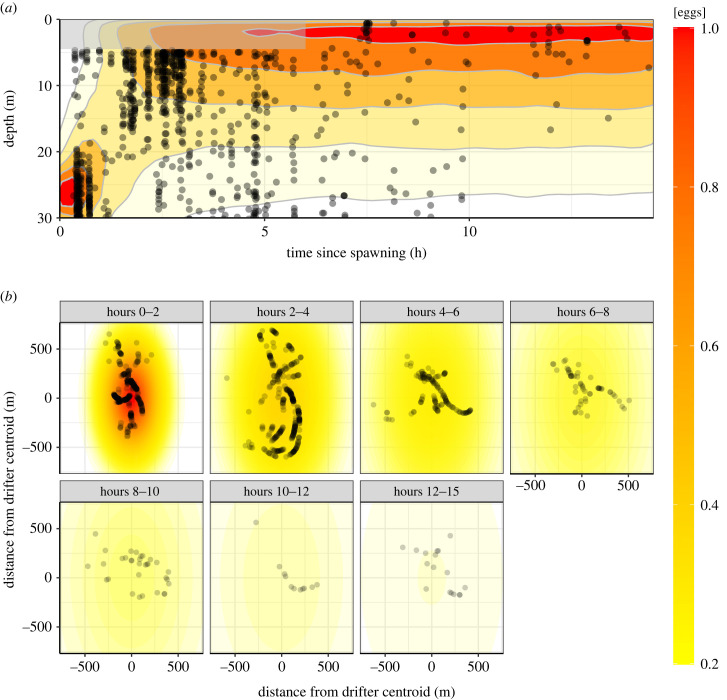
Observed vertical and horizontal distributions of individual Nassau grouper eggs around drifters released during spawning on 14 February 2017. Points indicate the location of images classified as Nassau grouper eggs: (*a*) time and depth, and (*b*) horizontal distance from the drifter centroid. Colour and contour lines show the predicted relative egg concentration from the three-dimensional diffusion–mortality model. Grey shading in (*a*) indicates depths and times that were not sampled.

By contrast, the two drifters released at the FSA during spawning on 14 February 2017 travelled off the shelf 18.3 km NNE over 14.5 h of *in situ* image sampling (electronic supplementary material, figure S1). The initial vertical egg distribution was approximately normal with mean depth 26.4 m and standard deviation 2.7 m. From 1–3 h after spawning, the eggs spread throughout the upper 30 m of the water column, and thereafter reached equilibrium concentrated in the upper 5 m (figure 3*a*). Horizontal diffusion was also evident, as the egg cloud increased in lateral extent and the concentration at the centre of the cloud decreased through time (figure 3*b*). The three-dimensional diffusion–mortality model explained much more of the deviance (24.2%, electronic supplementary material, figure S8 and table S2). Horizontal diffusivity was estimated to be *K_x_* = 14 900 m^2^ h^–1^ (95% CI: 12 000–19 000) = 4.1 m^2^ s^–1^ (95% CI: 3.3–5.3) in the east–west direction and *K_y_* = 49 100 m^2^ h^–1^ (95% CI: 40 800–60 500) = 13.6 m^2^ s^–1^ (95% CI: 11.3–16.8) in the north–south direction (electronic supplementary material, table S2). Mortality was estimated as *µ* = 0.172 h^–1^ (95% CI: 0.148–0.197), which implies that daily mortality was 4.13 day^–1^ and e^(−0.172×24)^ = 1.6% of the eggs survived to hatching at 24 h.

### (d) Modelled dispersal from spawning in previous years

**Figure 4.**
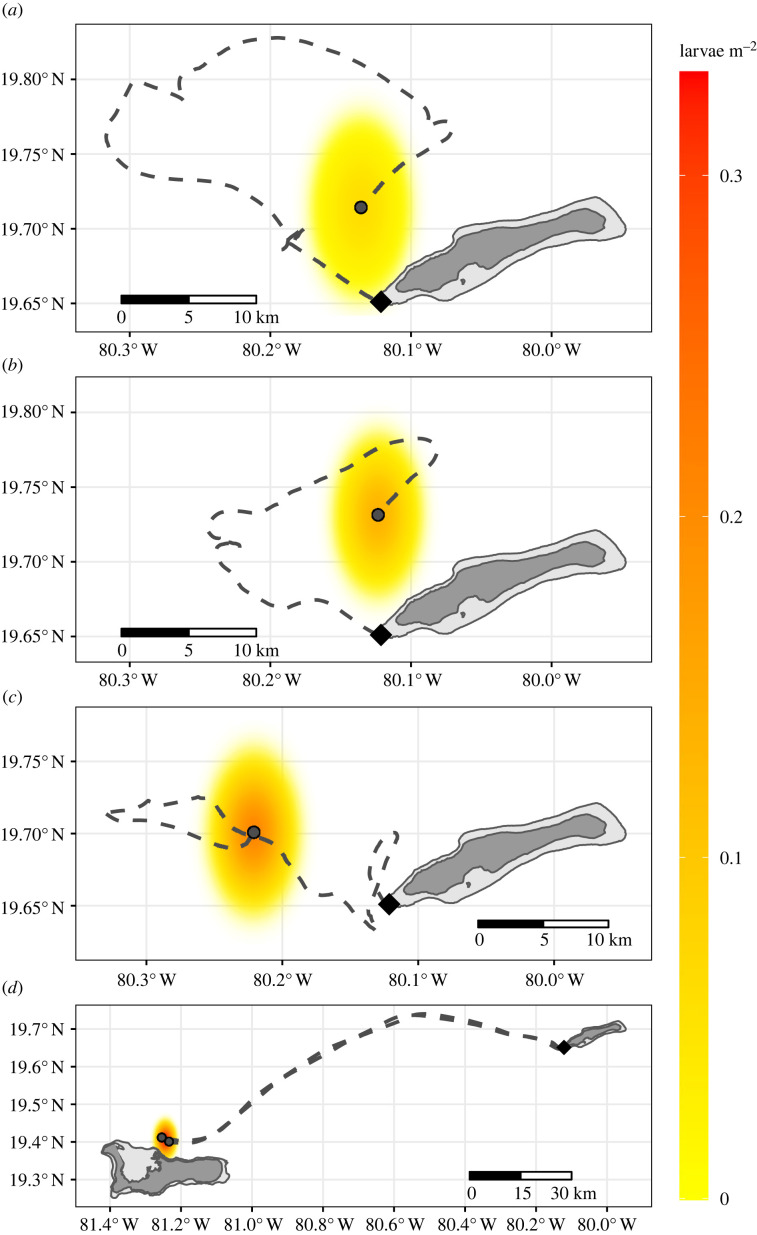
Estimated initial transport of larvae spawned at the Little Cayman aggregation in 2011 and 2016. Drifters (points) were released at the aggregation site (diamond) on nights of observed spawning: (*a*) 24 February 2011, 5 days later (4 days post-hatch, dph; *n* = 1); (*b*) 25 February 2011, 3 dph (*n* = 1); (*c*) 26 February 2016, 3.5 dph (*n* = 1); and (*d*) 25 February 2016, 2.9 dph (*n* = 2). The predicted concentration (yellow colour) is higher and less diffuse in (*b*) than (*a*) because less time has elapsed after spawning, and higher in (*c,d*) than (*b*) because the spawning biomass was higher in 2016 than 2011 and released roughly 2.4× more eggs. Light grey indicates 0–30 m depth, roughly the insular shelf extent.

Drifters released from the Little Cayman FSA on the two nights of peak observed spawning in 2011 travelled off the shelf but were within 10 km of the FSA 4–5 days later (figure 4*a,b*), i.e. 3–4 dph, when larvae exhaust their yolk reserves and begin actively feeding [55]. Applying the diffusion–mortality model estimated from 14 February 2017 data to these drifter tracks, we predict that high concentrations of larvae were retained on Little Cayman reefs in 2011. By contrast, drifters released on the two peak spawning nights in 2016 exhibited different behaviour. Applying the diffusion–mortality model to the 26 February 2016 drifter track indicates that either unknown and unmodelled currents, or substantially more active larval behaviour that favoured retention, would have been necessary for larvae to have dispersed back to Little Cayman (figure 4*c*). Two drifters released during spawning on 25 February 2016 moved much faster WSW, and our model predicted that passively acting larvae reached reefs on the north side of Grand Cayman at 3 dph (figure 4*d*).

## 4. Discussion

We leveraged emerging *in situ* imaging technology to reveal the biophysical processes driving early life history and recruitment dynamics of the largest remaining Nassau grouper spawning aggregation. We directly tracked one cohort of Nassau grouper eggs and yolk-sac larvae from the Little Cayman FSA back to Little Cayman reefs in 2017 (figure 2). Drifter tracks and a diffusion–mortality model showed that larvae from the Little Cayman FSA were also likely transported back to Little Cayman in 2011 and to Grand Cayman in 2016 (figure 4).

Although larval transport is thought to be a key process that affects recruitment variability [1–4], larvae must also successfully feed, avoid predation and find suitable reef habitat. The relative importance of these processes in controlling recruitment of Nassau grouper is unknown. However, initial transport of larvae away from the reef followed by a return, as observed for both peak spawning nights in 2011, is hypothesized to be evolutionarily advantageous for large reef fish because it reduces intense predation on eggs and larvae [72]. This hypothesis predicts high survival from the 2011 spawning, which is the same year that a population assessment estimates that a large cohort recruited to Little Cayman and drove a major population recovery [47]. Taken together, these results strongly suggest that the large 2011 year class self-recruited and that the Little Cayman population is self-replenishing.

### (a) Assumptions and limitations

Without direct observations, we cannot definitively conclude that the larvae produced at the Little Cayman FSA in 2011 and 2016 ultimately recruited to the Cayman Islands. Nevertheless, the dispersal model based on drifter tracks, diffusion and mortality strongly suggests that larvae reached Little Cayman in 2011 and Grand Cayman in 2016 (figure 4). Three assumptions underpin this interpretation: (1) diffusion and mortality rates estimated from 2017 observations apply in other years, (2) eggs and larvae are passive over the timescale considered (up to 4 dph), and (3) once larvae are passively transported to reefs, they remain near shore until they are ready to settle.

In essence, our simple dispersal model evaluated whether drifters coming ‘close’ to reefs were ‘close enough’ to transport larvae acting as passive particles, assuming the diffusion and mortality rates we estimated in 2017. Using these values to model dispersal in other years was conservative for two reasons: sea state was calmer in 2017 (diffusivity increases with sea state), and the 2017 diffusivity estimates were from shorter time and length scales (diffusivity increases with scale [73]). Thus, our estimates of diffusion around the 2011 and 2016 drifter tracks likely represent lower bounds on the extent of larval transport to reefs.

Another assumption of our analysis is that eggs and larvae were passive over the timescale considered (up to 4 dph). This is also reasonable, as the swim bladder and fins used later to influence transport are not yet developed in pre-flexion, yolk-sac larvae [45,46,74]. We would not, however, extrapolate the diffusion–mortality model with passive larvae beyond yolk-sac absorption and first feeding (4 dph).

Finally, our model is limited to 4 dph, yet the presumed pelagic larval duration (PLD) of Nassau grouper is 35–45 days. A combination of near-shore physics and larval behaviour would have been necessary for larvae to remain near reefs until they were ready to transition from the pelagic larval to the demersal juvenile stage. Larval behaviour, including vertical migration early in development by swim bladder control and later by active swimming, can take advantage of physical features to enhance retention [45]. Additionally, several local physical features occur in the vicinity (0–2 km) of reefs which can act to increase larval retention, such as reduced flow near shore (i.e. the ‘sticky’ boundary layer), tidal fronts, island wakes and Langmuir circulations [31,74–78]. Most biophysical dispersal models do not provide high enough spatial resolution to capture these complex near-shore processes, and instead, researchers subsume them with diffusive terms [79]. Models also differ in how to treat simulated larvae that reach suitable habitat before settlement stage. One approach is to assume that larvae stay and settle once they reach suitable habitat, given the retention mechanisms described above [79]. Based on this interpretation, the model results presented here suggest that larvae spawned at the Little Cayman FSA in 2011 and 2016 likely reached and settled on Caymanian reefs. This is further supported by studies showing that 2011 recruitment was a substantial driver of population recovery on Little Cayman [47,48,80,81].

The question of what happens to eggs and larvae passively transported to reefs well before settlement also applies to the cohort spawned on 15 February 2017, which we directly tracked back to the Little Cayman shelf (figure 2). The current paradigm of reef fish recruitment is that pelagically spawned eggs move offshore and develop away from the reef, and then larvae return after a significant duration. The hypothesis for the evolutionary motivation underlying this behaviour is that predation on eggs would be extremely high on coral reefs and lower offshore [72], consistent with several common characteristics of reef fish eggs that minimize predation: transparency, rapid development and spawning at sunset [72]. We would therefore expect the 15 February 2017 cohort to have experienced very high predation mortality. On the other hand, we would also expect larvae developing in the nutrient-rich coastal zone to grow faster and settle earlier, as well as experience little loss due to advection [6,23]. Since we did not sample settlement or juvenile stages, we are limited to future observations, e.g. length distributions, of adult spawners to tell whether a strong year class from 2017 spawning recruits to the Little Cayman FSA, as likely occurred in 2011 [47].

### (b) Utility of diffusion and mortality estimates

Field estimates of fish egg mortality are wildly variable, and we are not aware of any for pelagic-spawning tropical reef fish. Our estimated daily mortality rate, 4.13 day^–1^ (95% CI: 3.55–4.73), is just above a range of 48 estimates from temperate species (0.02–3.64 day^–1^) [82]. However, fish egg mortality increases with temperature, and the expected daily mortality at the temperature observed (27°C) during our sampling is $0.03e^{0.18\;\times \;27}=\;3.87\;{\mathrm{day}}^{\text{–}1}$ with 1 s.e. range of 2.26–6.64 [70]. Thus, our estimate of Nassau grouper egg mortality is above the average for temperate species, as expected, but within the expected range when taking temperature into account.

The horizontal diffusivities estimated here, 4–13 m^2^ s^–1^, are toward the upper end of the theoretically predicted range given the observed cloud size, approximately $3\sqrt{2\sigma_{x}\sigma_{y}}=300-1500\;m$ [73]. The higher horizontal diffusivity estimated in our study may be the result of vertical current shear, especially during initial dispersal when the egg cloud was at the edge of the Little Cayman shelf. Our diffusivity estimates are likely lower than Nassau grouper eggs typically encounter in the waters surrounding Little Cayman because the wind speed (2 m s^−1^) and sea state (less than 0.5 m, Beaufort 2) were anomalously calm in 2017, whereas conditions during spawning are usually in the range Beaufort 35. At higher wind speeds, eggs would also be more evenly distributed with depth.

### (c) Contribution of *in situ* imaging to studies of larval dispersal and recruitment

This study demonstrates how new observational platforms can further our understanding of physical–biological processes that determine the population dynamics of tropical reef fish. Our *in situ* imaging system provided sufficient optical resolution to document egg and larval development, even individual cells in the cleavage period (figure 1*a,b*), and verified that drogued drifters successfully track eggs from discrete spawning events. The system was deployed from a 14 m vessel without a winch, which lowered costs and increased manoeuvrability close to shore compared with using a large oceanographic research vessel typically required for *in situ* imaging studies. Critically, it also generated sufficient 3D ichthyoplankton position data to estimate diffusivity and mortality parameters in a biophysical dispersal model (figure 3; electronic supplementary material, tables S1 and S2), which allowed us to predict retention and export in other years (figure 4).

The capability to observe plankton at high temporal and three-dimensional spatial resolution via *in situ* imaging is rapidly evolving [83]. We envision that *in situ* imaging could shed light on a number of important physical–biological processes affecting larval fish dispersal and recruitment, including: (i) annual variation in diffusivity and mortality; (ii) *in situ* variability in egg and larvae development, growth and mortality, and how these compare with laboratory-based estimates that typically remove bacteria, parasites, predators and food limitation; (iii) spatio-temporal overlap with predator and prey distributions [84]; (iv) spatial variability or density-dependence in mortality; and ultimately, (v) separation of mortality into the three key processes governing recruitment success: predation, starvation and transport [1,85].

Despite the promise of imaging-based studies of larval dispersal, there are significant logistical challenges to consider:

(1) *Location and timing of spawning (initial condition/release site)*. We had detailed knowledge accumulated from 15 years of observations [44,50], as well as divers in the water to mark the egg plume and collect fertilized eggs. Many demersal species aggregate at predictable times and areas, and FSA sites can be located with fisher interviews, hydrophones, egg sampling and mapping catch of ripe fish [86]. Pelagic species may spawn over larger, less fixed areas but it is still possible to find and study egg and larval patches [87].
(2) *Classification of plankton images/identification of target organism*. In many cases fish eggs and early larvae are difficult or impossible to visually distinguish to species, even in the lab. We were fortunate that egg size, verified with DNA barcoding, was a reliable metric (electronic supplementary material, figure S3) and that the initial concentration was high at the mass spawning events. In other cases, more extensive sample collection and DNA barcoding may be necessary to supplement image classifications. Classifying large image datasets is also challenging, whether done manually or automated. However, improvements in machine learning and imaging technology will increasingly make this less of a limitation than the more fundamental issue of what can be visually identified *in vitro* [83].
(3) *Finding enough target organisms*. Imaging produces very high-resolution data but a big challenge is to sample enough target organisms to support analysis over ecologically relevant spatial and temporal scales. Over the PLD, larvae are expected to occur at vanishingly low concentrations from mortality and diffusion, and potentially increasing distances from shore. This limits how long into the PLD it is feasible to follow larvae patches. This challenge can be overcome with ingenuity and technological progress to enable sampling larger volumes of water. In addition, imaging systems will likely continue to decrease in size and cost [88,89]. This should facilitate deploying multiple imaging systems simultaneously from independent platforms, e.g. gliders, profiling floats, and saildrones [88,90–93]. The effective volume sampled can also be increased by concentrating plankton in the imaging field of view, e.g. with nets (electronic supplementary material, figure S2A), although net avoidance then becomes a concern for stronger-swimming organisms like late-stage fish larvae.
(4) *Nearshore physics*. Fish often spawn and settle at sites with complex physical features that occur below the resolution of most biophysical dispersal models (see *Assumptions and limitations*, §4a). Observing and modelling currents in three-dimensional and at higher spatio-temporal resolution near shore is therefore a challenge. However, it is also especially important for understanding the initial (spawning) and final (settlement) periods of dispersal, which are likely critical in determining successful recruitment [37].
(5) *Population-level effect and relevance to management*. Many studies of larval dispersal are conducted on small, model species at scales that are not directly relevant to fisheries management or conservation ([94], but see [7]). By contrast, Nassau grouper support fisheries and dive-based tourism, and we were able to place the observed dispersal in the context of stock assessment estimates of annual population size and recruitment [47]. Still, while the Little Cayman Nassau grouper FSA has substantial conservation and local economic value [61], the population is currently small and insignificant compared with those supporting large commercial fisheries, e.g. cod, tuna and herring. These larger stocks spawn over much larger spatial and temporal scales, making imaging-based studies of larval dispersal difficult. However, their high economic value may make some of the strategies listed above feasible, particularly using multiple non-ship-based platforms simultaneously.

Given these challenges, we suggest that one potential approach for studying the role of larval dispersal in determining year class strength is to combine (1) field observations of initial transport from spawning sites (as in this study) with (2) a biophysical dispersal model covering the majority of the PLD and (3) a second phase of larval sampling targeting the settlement period. Where and when to sample settlement-stage larvae could be informed by results from the physical dispersal model. Pairing these results with monitoring of juveniles or adult spawners would also allow stronger conclusions. In our case, we interpret favourable initial transport back to Little Cayman reefs following 2011 spawning in the light of observing many 1-year-old juveniles in 2012 [80,81] and a pulse of adults joining the spawning aggregation in 2017 [47].

### (d) Spawning aggregation protection in fisheries management

Fisheries that target spawning aggregations have proven particularly vulnerable to overfishing, and many species associated with these fisheries are now in peril, particularly in the tropics [95]. Thus, efforts to understand recruitment for these species typically focus on conservation and rebuilding from collapse. Our findings indicate that the largest remaining Nassau grouper spawning aggregation is self-replenishing, and the potential also exists for the export of larvae to sites in the surrounding region where only remnant populations exist. This is the ultimate goal of marine protected areas (MPAs) when considered as fisheries management tools: to sustain local populations through self-recruitment while also seeding recruitment and recovery in surrounding fished areas [11]. Two decades of spatio-temporal protection and monitoring on Little Cayman seem to have accomplished this goal for Nassau grouper, lending credence to calls to incorporate spawning aggregation protections into fisheries management [12,47,48,95].

## Data Availability

Data and code underlying this analysis are available at https://doi.org/10.5281/zenodo.6821663 [96]. Supplementary material is available online [97].
